# Linear epitope mapping of the humoral response against SARS-CoV-2 in two independent African cohorts

**DOI:** 10.1038/s41598-023-27810-1

**Published:** 2023-01-16

**Authors:** Inès Vigan-Womas, Jean-Louis Spadoni, Thomas Poiret, Fabien Taïeb, Fanirisoa Randrianarisaona, Rokhaya Faye, Adji Astou Mbow, Aboubacry Gaye, Ndongo Dia, Cheikh Loucoubar, Diary Juliannie Ny Mioramalala, Rila Ratovoson, Rindra Vatosoa Randremanana, Amadou Alpha Sall, Moussa Seydi, Josselin Noirel, Gabriel Moreau, Arnaud Simon, Pavlo Holenya, Jean-Philippe Meyniel, Jean-François Zagury, Matthieu Schoenhals

**Affiliations:** 1 Immunophysiopathology and Infectious Diseases Department, Institut Pasteur de Dakar, Dakar, Senegal; 2 Laboratoire Génomique, Bioinformatique, et Chimie Moléculaire, EA7528, Conservatoire National des Arts et Métiers, Hesam Université, Paris, France; 3 Epidemiology, Clinical Research and Data Science Department, Institut Pasteur de Dakar, Dakar, Senegal; 4 Institut Pasteur de Madagascar, BP 1274, 101 Antananarivo, Madagascar; 5 Virology Department, Institut Pasteur de Dakar, Dakar, Senegal; 6 Service des Maladies Infectieuses et Tropicales, Fann University Hospital Center, Dakar, Senegal; 7 Bioinformatics Team, Peptinov, Hôpital Cochin, 27 Rue du Fbg Saint-Jacques, 75014 Paris, France; 8 JPT Peptide Technologies GmbH, Berlin, Germany; 9 Bioinformatics Department, ISoft, Parc des Algorithmes, Bâtiment Euclide, Route de l’Orme, 91190 Saint-Aubin, France

**Keywords:** Antibodies, Immunology, Adaptive immunity, Infection, Infectious diseases

## Abstract

Profiling of the antibody responses to severe acute respiratory syndrome coronavirus-2 (SARS-CoV-2) proteins in African populations is scarce. Here, we performed a detailed IgM and IgG epitope mapping study against 487 peptides covering SARS-CoV-2 wild-type structural proteins. A panel of 41 pre-pandemic and 82 COVID-19 RT-PCR confirmed sera from Madagascar and Senegal were used. We found that the main 36 immunodominant linear epitopes identified were (i) similar in both countries, (ii) distributed mainly in the Spike and the Nucleocapsid proteins, (iii) located outside the RBD and NTD regions where most of the reported SARS-CoV-2 variant mutations occur, and (iv) identical to those reported in European, North American, and Asian studies. Within the severe group, antibody levels were inversely correlated with the viral load. This first antibody epitope mapping study performed in patients from two African countries may be helpful to guide rational peptide-based diagnostic assays or vaccine development.

## Introduction

The COVID-19 pandemic has been a shock with a major impact throughout the world. While only a fraction of the infected individuals declares a severe disease, this fraction is large enough to prevent the stable functioning of health structures in most countries. Despite the rapid development of efficient vaccines, the epidemic has remained a burden for the world with the regular emergence of new variants. Investigating specific immune responses in various populations in terms of origin and clinical profiles should allow us to better define the immune correlates of protection and help understand and improve effective diagnostic tests and vaccines adapted to local populations. In this line, several epitope mapping studies based on the recognition of linear peptide epitopes by patient sera have previously taken place in Asia^1–5^, in Europe^6–9^, and in North America^10,11^ in the last two years. To our knowledge, such a study has never been completed in African populations.

The goal of the present study was thus to explore in a systematic manner the humoral responses of African patients against the structural SARS-CoV-2 proteins of the envelope (E), the membrane (M), the nucleocapsid (N) and the spike (S) which are known primary targets of antibodies. We therefore used serum samples from patients with very well characterized disease evolution profiles (asymptomatic, symptomatic, and fatal cases) coming from two African countries with distinct ethnic origins, Madagascar and Senegal, and compared their 487 SARS-CoV-2 peptide-specific immunoglobulin levels (IgG and IgM) with those of sera collected in these countries prior to the year 2019. A panel of 36 peptides located mainly in S and N proteins displayed the highest antibody reactivities with 9, 2 and 25 peptides recognized by patients’ IgM, IgM/IgG and IgG respectively. The IgG immunodominant epitopes were the same as the ones previously described in Asian, European, or North American epitope mapping studies^1–11^. Taken together, the immune epitopes found in this study may help the development of epitope-based serological diagnostic assays or effective vaccine candidates^12^.

## Results

### Demographic and clinical information of the two independent COVID-19 African cohorts

COVID-19 positive individuals were recruited from two independent cohort surveys recruited in Madagascar and Senegal from the beginning of the pandemic in 2020. In Antananarivo, Madagascar, samples and data were collected from 16 infected individuals as part of the WHO’s First Few X cases (FFX) investigation protocol for COVID-19 (see Methods). In Senegal, SARS-CoV-2 infected patients (n = 66) were selected as part of a hospitalized cohort survey (see Methods). SARS-CoV-2 infection was confirmed in all the patients by qRT-PCR tests for SARS-CoV-2 nucleic acid in nasopharyngeal swabs. According to their clinical outcomes, the 82 SARS-CoV-2 infected individuals were divided into 3 main groups: asymptomatic (n = 28, 18 from Senegal, 10 from Madagascar), symptomatic (n = 28, 22 from Senegal, 6 from Madagascar), and severe (fatal clinical outcome) cases (n = 26). The latter group, composed only of Senegalese patients, died during their hospitalization.

The demographic, clinical, and group descriptions of the selected patients are summarized in Table 1. Included patients were mainly males (54.8%) with ages ranging from 11 to 83 years old (median age of Senegal and Madagascar was 57 and 46 years old, respectively). Patients from the Senegalese severe group were mainly males (72.0%) older than individuals from other groups (median 70 years old), with the highest viral loads (lowest cycle threshold (Ct), 30 cycles) and highest CRP levels (192 mg/ml) and leukocyte counts (14 × 10^9^ cells/L). None of the patient included in the study was affected by an active infectious disease such as HIV-1, Tuberculosis, or Malaria. These parameters were not collected in the Malagasy cohort and were thus not reported. Plasma samples (n = 42, 32 from Senegal, 10 from Madagascar) collected from healthy individuals before the emergence of SARS-CoV-2 in each country were included as controls in the peptide array analysis.

**Table 1:** Demographics and clinical characteristics of Senegalese and Malagasy patients.

	Senegal			Madagascar*	
	Asymptomatic	Symptomatic	Severe	Asymptomatic	Symptomatic
Population	18	22	26	10	6
**Sex**					
Male	9	10	18	4	4
Female	9	12	8	6	2
Age Median	41 (11–73)	53 (21–74)	70 (53–83)	41 (21–57)	66 (51–75)
RT-PCR Ct values Median	33 (26–39)	34 (26–38)	30 (14–37)	NA	NA
CRP (mg/ml) Media	6 (1–10)	8 (3–165)	192 (24–219)	NA	NA
WBC count (10^9^ cells/L) Median	4.85 (3.4–8.1)	5.5 (2.8–27.2)	14 (6–144)	NA	NA

Numbers correspond to the median with the min–max span in parenthesis.

RT-PCR, SARS-CoV-2 real-time PCR cycle threshold (Ct) values; CRP, C-reactive protein; WBC, White blood cells.

*In Madagascar, the biological parameters (viral load, CRP, and WBC) were not available.

### Mapping of the anti-SARS-CoV-2 humoral response in infected and uninfected individuals

Sera obtained within 2 weeks after patient hospitalization were tested by peptide microarrays designed to present 487 overlapping peptides (15-mers with 11 amino acid overlap) from the 4 structural SARS-CoV-2 proteins E, M, N, and S (listed in suppl. Table 1). Peptide-specific IgG and IgM levels were evaluated for each individual. After data cleaning (see Methods), the values obtained for each peptide were compared between the 42 uninfected and the 82 SARS-CoV-2 infected people.

**Figure 1:**
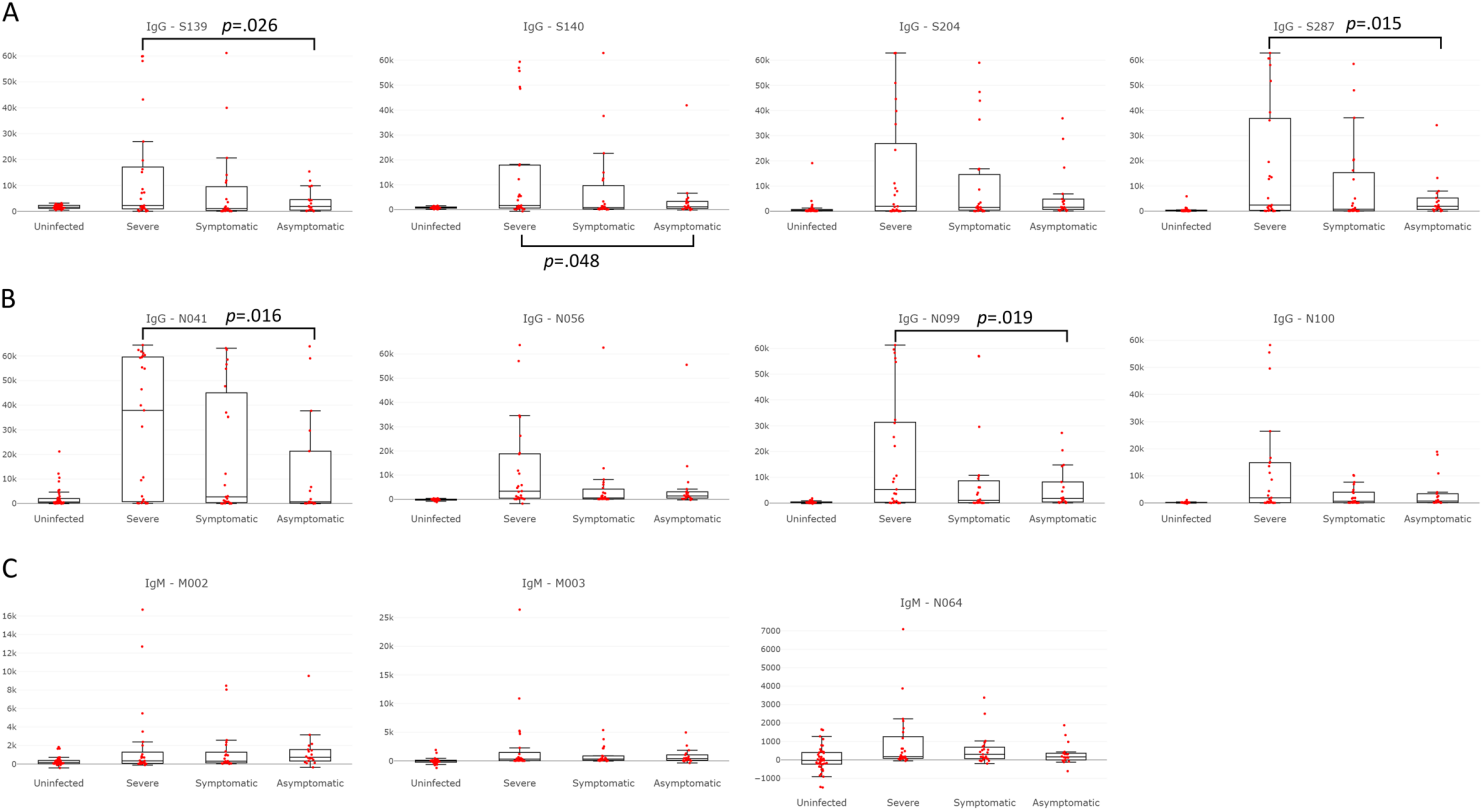
IgG and IgM specific responses against the most significant immunodominant linear epitopes within different clinical groups. Responses against the most significant peptides highlighted in bold in Table 2. (**A**) IgG responses toward spike peptides S139, S140, S204 and S287. (**B**) IgG responses toward nucleocapsid peptides N041, N056, N099 and N100. (**C**) IgM response toward membrane peptides M002 and M003 and nucleocapsid peptide N064. *p* values for the comparison of uninfected versus infected subjects are all significant for the chosen peptides of Table 2. The *p* values for the comparison of the asymptomatic group versus the symptomatic group were always non-significant. Here, *p* values are shown under the graphs only if the comparison between the severe group and the asymptomatic groups by the Student's t-test is significant.

A stringent statistical test identified eight peptides significantly higher in IgG *p* value in infected individuals (*p* < 3 × 10^−4^): 4 in the protein S (S139, S140, S204 and S287) and 4 in the protein N (N41, N56, N99 and N100) (Table 2). IgM specific responses also showed 4 peptides with significantly higher *p* value in infected individuals as compared to healthy ones: 2 in the protein M (M002 and M003), 1 in the protein N (N64), and 1 in the S protein (S287) which also displayed a high IgG reactivity. When looking at the detailed humoral responses for these 12 immunodominant peptides across the 4 different groups of individuals, the strongest antibody responses toward those peptides were seen mostly in severe cases as shown in Fig. 1. A less stringent analysis of our data to obtain a broader list of immunodominant peptides identified a total of 39 out of 487 SARS-CoV-2 peptides screened that bound significantly more (Table 2). These peptides include 27 and 12 peptides respectively with higher IgG or IgM *p* value in the infected group than in the uninfected one. We observed 2 peptides exhibiting both a strong IgM and IgG response: N091 and S287. As shown in Table 2, all the peptides recognized by IgG had also been identified by the previous epitope mapping studies^1–11^. The antibody specificities predominantly targeted 3 main antigenic regions in the N protein, 6 in the S protein and one in the membrane domain. Indeed, several clusters of overlapping peptides that share linear sequence identity of at least 7 amino acids for IgGs (N40-N41-N42, N97-N98-N99-N100, S139-S140, S165-S166, S197-198, S202-S203-S204, S287-S288, S313-S314-S315) as well as for IgMs (M002-M003, N063-N064) humoral responses were identified (Table 2).

**Table 2:** List of the immunodominant linear B cell peptides.

Peptide name	Peptide localization	Peptide start	Peptide end	Sequence	Ig type	Previous references
N010	Nucleocapsid	37	51	SKQRRPQGLPNNTAS	IgG	^7^
N040	Nucleocapsid	157	171	IVLQLPQGTTLPKGF	IgG	^4,7,8,10^
**N041**	Nucleocapsid	161	175	LPQGTTLPKGFYAEG	IgG	^4,7,8,10,11^
N042	Nucleocapsid	165	179	TTLPKGFYAEGSRGG	IgG	^4,8,10,11^
**N056**	Nucleocapsid	221	235	LLLLDRLNQLESKMS	IgG	^2,4,7,11^
N058	Nucleocapsid	229	243	QLESKMSGKGQQQQG	IgG	^2,11^
N094	Nucleocapsid	373	387	KKKADETQALPQRQK	IgG	^2,4,7,10,11^
N097	Nucleocapsid	385	399	RQKKQQTVTLLPAAD	IgG	^2,4,7,10,11^
N098	Nucleocapsid	389	403	QQTVTLLPAADLDDF	IgG	^2,4,7,10,11^
**N099**	Nucleocapsid	393	407	TLLPAADLDDFSKQL	IgG	^2,4,7,10,11^
**N100**	Nucleocapsid	397	411	AADLDDFSKQLQQSM	IgG	^2,4,7,10,11^
**S139**	Spike	553	567	TESNKKFLPFQQFGR	IgG	^1–3,5–7,10,11^
**S140**	Spike	557	571	KKFLPFQQFGRDIAD	IgG	^1–3,5–7,10,11^
S165	Spike	657	671	NNSYECDIPIGAGIC	IgG	^1,4,6,7,9^
S166	Spike	661	675	ECDIPIGAGICASYQ	IgG	^1,5,6^
S195	Spike	777	791	NTQEVFAQVKQIYKT	IgG	^6,11^
S197	Spike	785	799	VKQIYKTPPIKDFGG	IgG	^2,6,7,11^
S198	Spike	789	803	YKTPPIKDFGGFNFS	IgG	^2,6,7,11^
S202	Spike	805	819	ILPDPSKPSKRSFIE	IgG	^1,2,6,7,10^
S203	Spike	809	823	PSKPSKRSFIEDLLF	IgG	^1,2,6,7,10,11^
**S204**	Spike	813	827	SKRSFIEDLLFNKVT	IgG	^1,2,4–7,10,11^
**S287**	Spike	1145	1159	LDSFKEELDKYFKNH	IgG	^1,2,6,7,10,11^
S288	Spike	1149	1163	KEELDKYFKNHTSPD	IgG	^1,2,6,10,11^
S313	Spike	1249	1263	SCGSCCKFDEDDSEP	IgG	^7,11^
S314	Spike	1253	1267	CCKFDEDDSEPVLKG	IgG	^7,11^
S315	Spike	1257	1271	DEDDSEPVLKGVKLH	IgG	^7,11^
N091	Nucleocapsid	361	375	KTFPPTEPKKDKKKK	IgG	^2,11^
**M002**	Membrane	5	19	NGTITVEELKKLLEQ	IgM	^2,10^
**M003**	Membrane	9	23	TVEELKKLLEQWNLV	IgM	^2^
N091	Nucleocapsid	361	375	KTFPPTEPKKDKKKK	IgM	
N063	Nucleocapsid	249	263	KSAAEASKKPRQKRT	IgM	^7^
**N064**	Nucleocapsid	253	267	EASKKPRQKRTATKA	IgM	
N093	Nucleocapsid	369	383	KKDKKKKADETQALP	IgM	
S007	Spike	25	39	PPAYTNSFTRGVYYP	IgM	
S012	Spike	45	59	SSVLHSTQDLFLPFF	IgM	
S033	Spike	129	143	KVCEFQFCNDPFLGV	IgM	
S085	Spike	337	351	PFGEVFNATRFASVY	IgM	
S120	Spike	477	491	STPCNGVEGFNCYFP	IgM	
**S287**	Spike	1145	1159	LDSFKEELDKYFKNH	IgM	^7^

The most significant peptides found by a stringent statistical test (*p* value < 3 × 10^−4^) are shown in bold. Common amino acids between overlapping peptides are underlined. The last column indicates the previous epitope mapping studies in Asian, European, and USA studies that found the same peptidic regions.

**Figure 2:**
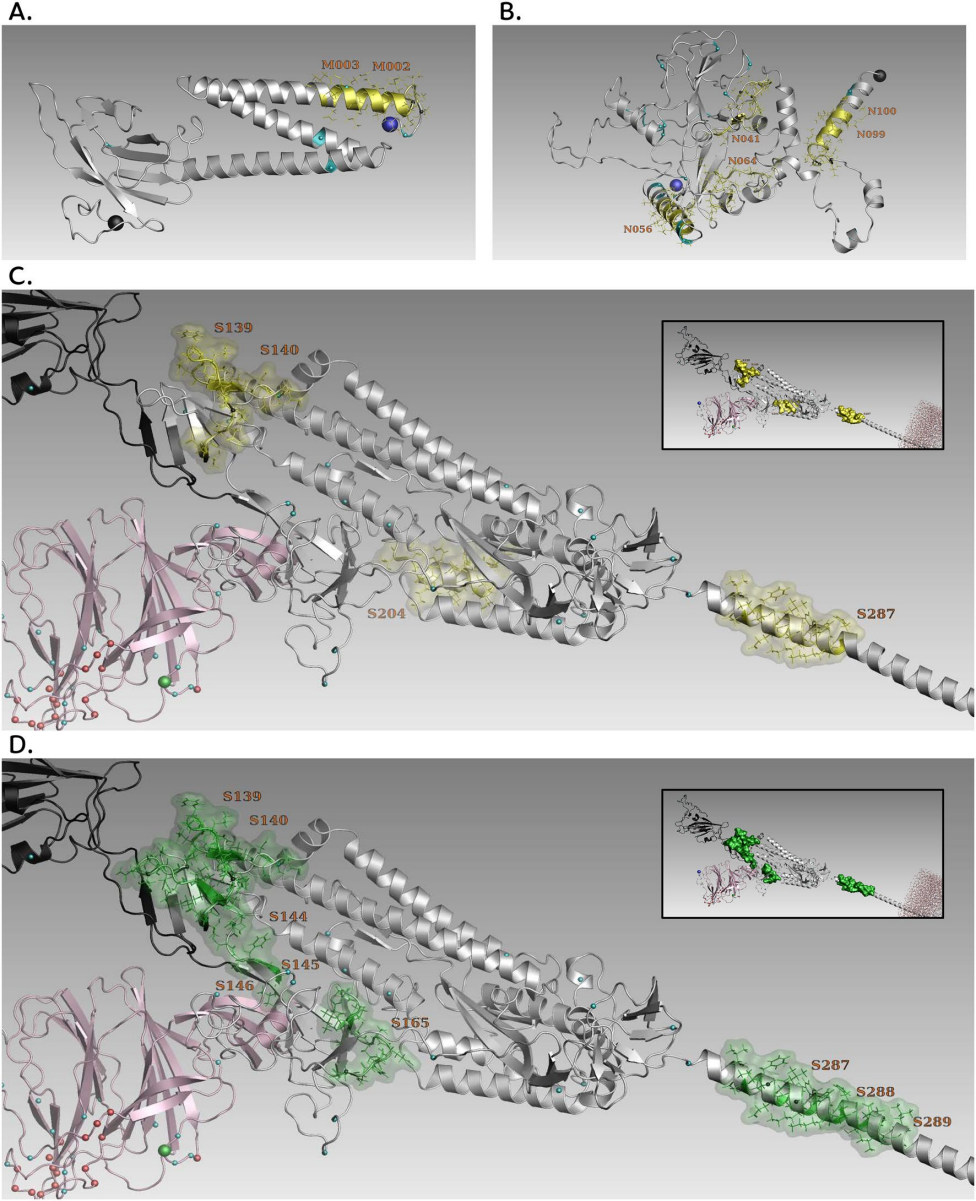
3D structure of the Spike, Nucleocapsid, and membrane proteins with locations of specific immunodominant epitopes. For all proteins, the blue spheres marked the N-Term extremity of the protein, black ones the C-term extremity, small light blue spheres indicated the main mutations found in SARS-CoV-2 variants described by WHO, small red spheres shown deletions, green spheres marked insertions, and the yellow segments corresponded to the immunodominant peptides found in our study (peptides marked in bold in Table 2). (**A**) The membrane protein with peptides M002 and M003 recognized by IgM. (**B**) The nucleocapsid protein with peptides N041, N056, N099 and N100 recognized by IgG and peptide N064 recognized by IgM. (**C**) Spike with peptides S139, S140, S204 and S287 recognized by IgG. RBD and NTD domains are represented in black and pink respectively. The main mutations in Spike are found in RBD and NTD. (**D**) representation of the spike protein with the neutralizing epitopes identified in previous epitope-mapping studies^1,5,6^ which are marked in green.

We used 3D models for the SARS-CoV-2 monomeric spike structure, the nucleoprotein structure, and the membrane protein structure, to show the location of the epitopes most strongly recognized by IgGs (Table 2), together with the main mutations recorded during the emergence of Alpha, Delta, Lambda and Omicron variants (Fig. 2). For Spike, we can see that the main immunodominant public epitopes identified in this study were rather well conserved and localized outside the highly mutational zones found mainly in the receptor-binding (RBD) and the N-terminal domains (NTD).

To gain a deeper insight into antibody responses in SARS-CoV-2 infected patients, we then looked at the differences in immunodominant peptides found in infected patients as compared to uninfected individuals in Senegal and Madagascar. There were significantly more peptides recognized by IgMs in the infected group from Madagascar than from Senegal (27 vs. 4 peptides, *p* = 0.006, Table 3). Nevertheless, for IgG reactivities, 9 out of 19 immunodominant peptides identified were common to both countries (Table 3).

**Table 3:** Peptides recognized by the Senegalese and Malagasy patient samples.

	IgG-recognized peptides (nb)	IgM-recognized peptides (nb)	IgG-/IgM- recognized peptides (nb)
**Infected versus uninfected**			
Senegal	**M002**, N040, N041, **N091**, N094, N097, N098, **N099**, **N100**, **S139**, **S140**, S203, **S204**, **S287**, **S288**, S314 **(16)**	**M002**, N091, N093, **S287** **(4)**	**M002**, N091, **S287** **(3)**
Madagascar	**M002**, M037, M050, **N091**, **N099**, **N100**, S088, S113, S114, S138, **S139**, **S140**, S157, S197, S198, S202, **S204**, **S287**, **S288** **(19)**	E015, **M002**, M008, M025, M030, M043, M049, N020, N021, N040, S007, S046, S051, S059, S060, S081, S086, S103, S112, S113, S139, S168, S236, S241, S268, **S287**, S288 **(27)**	**M002**, S113, S139, **S287**, S288 **(5)**
**Senegal**			
1. Severe versus uninfected	N010, N040, N041, N042, N058, N091, N094, N097, N098, N099, N100, S139, S140, S165, S166, S197, S198, S202, S203, S204, S287, S288, S314, S315 (24)	M003, N063, N064, N091, N093, S007, S020, S033, S275, S287 (10)	N091, S287 (2)
2. Symptomatic versus uninfected	N040, N041, N090, N099, N100, S139, S140, S203, S204, S287, S288, S295 (12)	M002, N091, N092, N093, S287 (5)	S287 (1)
3. Asymptomatic versus uninfected	M002, N040, N041, N058, N098, N099, N100, S140, S197, S203, S204, S287 (12)	M002, N022, N035, N091, N093, S287 (6)	M002, S287 (2)
4. Severe versus asymptomatic	N010, N040, N041, N042, N056, N063, N065, N088, N091, N093, N094, N097, N098, N099, N100, S006, S007, S010, S017, S039, S061, S062, S139, S140, S165, S166, S195, S203, S204, S287, S288, S313, S314, S315 (34)	N063, N064, S007, S012, S014, S033, S034, S046, S085, S086, S112, S120, S138, S202, S273, S274, S275, S301 (18)	N063 (1)
5. Symptomatic versus asymptomatic	N009, N040, N041, N090, N091, S095, S203, S204, S287, S288, S289, S295 (12)	N091, N092, N093, S008 (4)	N091 (1)
6. Severe versus symptomatic	E013, E015, M025, M026, M043, M044, M047, M048, M049, M050, N010, N019, N028, N042, N056, N058, N059, N063, N065, N067, N073, N074, N088, N091, N093, N094, N097, N098, N099, N100, S006, S007, S008, S009, S010, S015, S017, S020, S024, S034, S035, S037, S039, S041, S060, S061, S062, S085, S086, S087, S088, S089, S092, S122, S123, S139, S140, S165, S166, S189, S195, S196, S197, S198, S202, S249, S259, S266, S271, S286, S287, S288, S302, S313, S314, S315 (76)	N010, N013, N036, N067, N069, N078, N102, S002, S014, S015, S019, S020, S023, S024, S025, S033, S045, S046, S053, S085, S088, S112, S120, S138, S140, S164, S202, S224, S273, S274, S275 (31)	N010, N067, S202 (3)
7. Asymptomatic versus symptomatic	E013, M025, M026, M036, M037, M043, M047, M048, M049, M050, N021, N026, N027, N028, N058, N059, S009, S034, S035, S041, S049, S050, S060, S066, S067, S085, S086, S087, S088, S093, S112, S189, S197, S198, S249, S265, S266, S301 (38)	N002, N003, N004, N005, N010, N031, N034, N035, N042, N051, N066, N067, N069, N076, S006, S019, S046, S047, S068, S148, S161, S199, S224, S270, S272, S283, S290, S307 (28)	/ (0)
8. Asymptomatic versus severe	M036, S265 (2)	M048, N002, N005, N022, N034, N042, N051, N097, S004, S075, S095, S105, S148, S154, S167, S190, S199, S269, S272, S283, S289, S290, S307, S309, S310, S311 (26)	/ (0)
9. Symptomatic versus severe	S289, S295 (2)	M048, N091, N092, N097 (4)	/ (0)

Senegal versus Madagascar comparison: peptides recognized both by Senegalese and Malagasy patients are highlighted in bold. For each comparison, the number of recognized peptides is indicated in brackets.

### Comparison of the anti-SARS-CoV-2 humoral responses between groups of patients

Using the same approach as above, we then investigated the differences in peptide-specific humoral responses between the three COVID-19 Senegalese patient groups: asymptomatic, symptomatic and severe, and the uninfected individuals (Table 3). For the comparison of the 3 infected groups with the uninfected group, most of the epitopes were found for the comparison of IgG specificity between the severe and the uninfected groups (line 1, Table 3). Most epitopes recognized by the 2 immunoglobulins found in symptomatic versus uninfected or in asymptomatic versus uninfected were identical to the ones in severe versus uninfected (lines 1, 2 and 3, Table 3). Severe versus symptomatic B cell peptide response comparison revealed the identification of the larger panel of peptides differentially recognized both in terms of IgG and of IgM, suggesting that symptomatic individuals were less responsive to SARS-CoV-2 antigens (lines 4 and 9, Table 3). Severe patients had many IgG and IgM peptides more recognized than in the symptomatic or asymptomatic individuals (lines 4 and 6, Table 3). Asymptomatic individuals had higher IgG and IgM recognized peptides than symptomatic patients, as shown by the comparison of the two groups (lines 5 and 7, Table 3). We also observed that asymptomatic individuals recognized globally more IgM-specific epitopes than severe patients (lines 4 and 8, Table 3). Reciprocally, the comparison of severe patients with asymptomatic individuals showed a shift towards more IgG-specific epitopes recognized (lines 4 and 8, Table 3).

Overall, we observed that (i) symptomatic individuals had less peptides recognized and smaller Ab responses (both IgM and IgG) than asymptomatic and severe cases, (ii) number of recognized epitopes by IgM was relatively more important in asymptomatic individuals witnessing the kinetics of an earlier stage of infection, and (iii) the number of IgG-specific epitopes was relatively more important in severe patients suggesting a longer infection period or a higher viral load exposure. Last, the presence of IgG-specific peptides in asymptomatic individuals compared to uninfected (line 3, Table 3) suggesting that some IgG may have been produced rapidly after infection^8,9,11^.

### Relationship between immune responses and biological parameters

**Figure 3:**
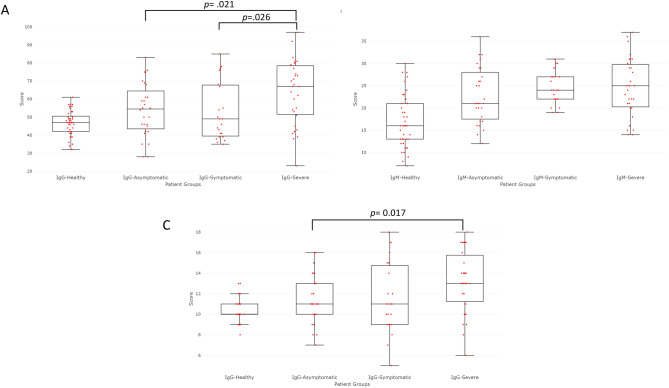
IgG and IgM scores against SARS-CoV-2 immunodominant peptides. Box-plot representation of the Ig scores of the patients against various peptides for each group. (**A**) IgG computed scores from the immunodominant peptides of Table 2. (**B**) IgM computed scores from the immunodominant peptides of Table 2. (**C**) IgG computed scores from the known neutralizing epitope peptides. For (**A**, **B**) the scores of the uninfected subjects are all significantly lower than the scores of the infected groups. In the (**A**–**C**) the scores of the severe group patients are always higher than the scores of the asymptomatic and symptomatic group, and the *p* values are shown under the graphs only if the comparison between two infected groups is significant by the Student's t-test.

Previous studies have described an amplified and broader response in severe patients compared to asymptomatic ones (1, 2). We computed for each patient a score measuring its global response (see Methods) against the SARS-CoV-2 public epitopes presented in Table 2 and found higher IgG and IgM scores for the patients in the severe group (Fig. 3). Interestingly, the IgG and IgM scores showed some level of correlation within the severe and within the symptomatic groups (corr = 0.5). This correlation was not observed in the asymptomatic nor in the uninfected groups (corr < 0.15). The lack of correlation in the asymptomatic group could be explained by an earlier stage of infection with lower IgG levels at the time of sampling.

In the Senegalese cohort, biological parameters available (including viral loads, CRP levels, granulocyte counts) were compared within severe and symptomatic group and between patients with high versus low IgG or IgM scores (i.e. analysis based on discrimination between individuals with a score higher or lower than the median, see Methods). Within the severe group, the patients with low IgG scores presented a significantly higher viral load (lower CT) as compared to the patients with a high IgG score (*p* = 0.02, Table 4). A similar difference was observed between low and high IgM scores. Interestingly, although non-significant, severe patients with higher antibodies (and thus lower viral loads) exhibited higher CRP levels and granulocyte counts: as if the immunological high responders (in terms of B cell response) were the ones who developed higher inflammatory markers, and vice-versa. Individuals from the symptomatic group presented in general lower CRP levels and granulocyte counts than those from the severe patients’ group (Table 4). Within the symptomatic group, no difference in viral load was observed between the high and low antibody level subgroups.

**Table 4:** Relationship between antibody scores and biological parameters in Senegalese patients.

	IgG score	IgM score	Viral load (nb PCR cycles)	CRP	Granulocyte count
**Sorted by the median of the IgG score against Table ****2**** peptides**					
A. Severe low IgG score	1.6 (1)	1.5 (1)	**27 (23)**	148 (164)	12.5 (13.8)
B. Severe high IgG score	8 (8)	4 (4)	**35.6 (36)**	178 (192)	14.9 (16.7)
C. Symptomatic low IgG score	1 (1)	0.8 (1)	36 (35.4)	23 (5)	6.2 (5)
D. Symptomatic high IgG score	4.7 (5)	3 (3)	35 (35)	48 (20)	7.9 (7.1)
**Sorted by the median of the IgM score against Table ****2**** peptides**					
A. Severe low IgM Score	2.9 (2)	0.5 (0)	**27.6 (28)**	145 (139)	12.3 (13.8)
B. Severe high IgM score	6.6 (7)	5 (4.5)	**36 (40)**	189 (195)	15.6 (16.7)
C. Symptomatic low IgM Score	1.5 (1)	0.4 (0)	36 (36)	23 (5)	5.7 (4.9)
D. Symptomatic high IgM score	4.2 (5)	3.3 (3)	34.7 (34)	54 (20)	8.5 (7.3)

The values given in the table were expressed as mean (median). In bold, a significant difference computed by the Student’s t-test (*p* < 0.05).

### Relationship between IgM and IgG epitopes

In our study, asymptomatic individuals exhibited more IgM peptide epitopes than other patient groups. This observation may be due to an earlier stage of infection and/or a more effective first-line humoral response profile than symptomatic patients. Some degrees of correlation between the IgM and IgG responses toward specific peptides were observed in the severe group of patients, probably because they could mount a mature humoral response with both, IgM and IgG. We then investigated whether several epitopes identified in the early stages of infection (IgM specific epitopes) were found in the later stages of infection (IgG specific epitopes) irrespectively of the patient groups. The epitopes that are commonly immunodominant for IgM and IgG were N091, S113, S139, S288, M002 and S287 found either in Senegal or in Madagascar with M002 and S287 however found in both sites (Table 3). In the Senegalese cohort, some epitopes initially observed in asymptomatic individuals (IgM response) were found at a later stage in severe patients (IgG response): N091 and S287 peptides (both in column 2/line 3 and in column 1/line1 of Table 3), but also N042, N097 (both in column 2/line 8 and in column 1/line1 of Table 3), and N093 peptides (both in column 2/line 3 and in column 1/line4 of Table 3). No peptides specific to the severe group and absent from the asymptomatic group, nor peptides specific to the asymptomatic group and absent from the symptomatic and severe groups were observed in our cohort.

### Neutralizing epitopes

An initial goal of our study was to investigate if some peptide epitopes were associated with resistance/susceptibility to infection or disease severity. As shown in Table 3, we have identified some correlations between some immunodominant epitopes and the asymptomatic, symptomatic, or severe phenotypes, but no causal relationship could be established. Among the previous epitope mapping studies cited^1–11^, three studies have described immunodominant neutralizing epitopes in Spike protein and confirmed them experimentally^1,5,6^. These confirmed neutralizing epitopes were peptides S554-573, S574-593, and S1146-1165 described by Yi et al.^1^, peptides S562-579 (fusion peptide) and S818-835 (close to RBD) described by Poh et al.^5^, and the peptide S655-672 described by Farrera-Soler et al.^6^. These peptides correspond to 4 regions of Spike covered by 13 peptides in our study: the first region corresponds to our peptides S139, S140, S141, S144, S145 and S146, the second region to our peptide S165, the third region to peptide S204, S205, and S206, the fourth region to our peptides S287, S288 and S289. These peptide regions of Spike are presented in Fig. 2D. We can observe that among those previously published neutralizing peptide epitopes, 6 peptides were found to be also immunodominant in our study (see Table 2), namely peptides S139, S140, S165, S204, S287, and 288.

Response against the 13 peptides covering these 4 published neutralizing regions were compared between of the 4 groups of subjects (Supplementary Fig. 1). Expectedly, the highest IgG response was found in severe subjects and the lowest in uninfected individuals as for the other immunodominant peptides of Table 2. We also established a cumulative score of response against these neutralizing regions (see “Methods” section, Fig. 3C). This cumulative score was higher in the three infected groups compared to the uninfected group. There was a significant difference between the scores of the severe group and the ones of the asymptomatic group, based on these potentially neutralizing peptides (Fig. 3C).

## Discussion

We have completed the first systematic study of the humoral responses against the 4 main SARS-CoV-2 structural proteins by epitope mapping in infected patients from Africa: 65 infected patients and 32 pre-pandemic serum controls from Senegal, 16 patients and 10 pre-pandemic controls from Madagascar. Our initial goal was to determine the main B cell epitopes recognized by patients and compare them with previous studies performed in Asia, Europe, or USA patients^1–11^, to see if distributions of IgM and IgG epitopes could explain the various COVID-19 clinical profiles.

Several immunodominant epitopes were identified for both, IgM and IgG. The responses observed in Madagascar and in Senegal were very similar with some differences that could be explained by the patient profiles (early infection vs. later infection stages) and possibly fluctuations linked to the smaller size of the Malagasy group. There was no immunodominant epitopes detected in the E protein as in previous studies^1–11^. All the IgG epitopes found in this study were similar to other earlier epitope mapping studies from Asia, Europe or the USA, indicating that they undoubtedly represent immunodominant epitopes. Several IgG epitope regions have been identified by no less than 5 different studies, in the nucleocapsid (N41, N94, N97, N98, N99) and in Spike (S139, S140, S165, S202, S203, S204, S287, S288) domains. Altogether, this confirms that the landscape of the specific humoral response of African patients against SARS-CoV-2 virus is very similar to that observed in other continents revealing that the diverse immunogenetic backgrounds of these populations do not significantly modify the humoral responses against SARS-CoV-2 linear peptides. Regarding the IgM epitopes, fewer epitopes were retrieved in common with other studies. Indeed, only a few epitope mapping studies have addressed IgM peptide epitopes, and few IgM epitopes have been described. Of note, the blood sampling was performed at a rather early stage in our study (mean at 10–12 days post onset of symptoms), which could explain the relatively strong IgM responses.

The immunodominant IgG epitopes obtained by epitope mapping were mainly localized outside the RBD and NTD regions of Spike that concentrate most of the known mutations of SARS-CoV-2. Similar observations were indeed made by many of the previous epitope mapping studies in Europe, Asia, or the USA^1–3,6,7,10^. Interestingly, among the immunodominant peptide epitopes identified in Table 2, some peptide regions have been proven to be targets of neutralizing antibodies by previous studies^1,5,6^, namely peptides S139, S140, S165, S204, S287, S288.

We were able to see that the IgM/IgG profile of the immune response was rather linked to the patient status at the time of blood sampling, with a shift towards more IgM epitopes for asymptomatic individuals, and a shift towards more IgG epitopes for severe patients who were likely more exposed to viral replication. Using the score of response against the SARS-CoV-2 group-specific peptide immunodominant epitopes, we could see that globally the severe group individuals exhibited higher scores of IgG and IgM responses than the asymptomatic or symptomatic groups, witnessing a more intense immune response for such patients as described by previous studies^1,11^.

We have observed that severe patients had more anti-SARS-CoV-2 antibodies than symptomatic patients. The difference of response intensity between severe and symptomatic patients is unlikely to be due to a longer infection period prior to blood collection for the severe patients, as the number of days between hospitalization and blood collection is 11 for the symptomatic group and 13 for the severe group. A more likely explanation could be that symptomatic people have indeed less antibodies than severe patients because the latter have been exposed to a more active viral replication. Conversely, we have observed that asymptomatic individuals have more antibodies than symptomatic ones that may be sufficient for neutralizing viral replication, while symptomatic individuals have a lower immune response and thus a higher viral load and more symptoms. Importantly, we observed that some individuals in the severe group had no longer detectable viral load at the time of blood sampling, and that severe patients with a higher score of antibodies had a lower viral load than severe patients with lower score of antibodies suggesting their humoral response may have contributed to eliminate the virus but did not enable their survival. Patients from the severe group were clearly older than the other groups and exhibited higher CRP levels and higher viral loads than that of the symptomatic group, showing a high sensitivity to COVID-19.

The role of the T cell, not measured in the present study, is critical to the general immune response against SARS-CoV-2 and could explain why the infection was suppressed in symptomatic individuals even in the presence of an imperfect humoral response^13–15^. Interestingly, a study has shown that the humoral response against seasonal viruses was weakened with susceptibility to COVID-19 and age, while the humoral response against Herpes virus family was increased^11^. We do know that T cell responses are critical in combating the fatal evolution of COVID-19 disease, as evidenced by previous research^16^ and the success of vaccines in limiting fatal cases. The lack of T cell response necessary to quell the inflammatory consequences of a persistent viral infection could explain why some severe patients, while mounting a suitable humoral response, did not survive. It will be interesting to assess the cellular immune response in these groups of patients. Additional factors such as co-morbidities or the genetics of the individuals may also make the patients of the severe group more susceptible to developing a fatal evolution following a persistent infection.

Overall, our study shows that the humoral responses against SARS-CoV-2 in patients from two African countries targets the very common linear epitopes described in previous studies performed in patients from Asia, Europe, and the USA^1–11^. Additional studies focusing on the peptides recognized by T cells should also be performed to get a broader view of the role of the T cell immune response in controlling viral infection and disease development.

## Methods

All methods used in this manuscript were performed in accordance with the relevant guidelines and regulations.

### Study populations and ethical approval statement

For Senegal, pre-pandemic samples (n = 32) were from a longitudinal cohort survey performed in Dielmo since 1990^17,18^. For this study, plasma samples obtained during the cross-sectional survey of June 2018 were used. The retrospective use of these samples for immunological analysis in the context of COVID-19 has been approved by the Senegalese National Ethics Committee for Research in Health (reference number 00000007/MSAS/CNERS/Sec 26 January 2021) and villagers have given their individual consent for this purpose. Samples from COVID-19 RT-PCR positive patients were obtained from a multicentric non-interventional national cohort survey, named SEN-COV^19^, approved by the Senegalese National Ethics Committee for Research in Health (reference number 00000068/MSAS/CNERS/Sec, 10 April 2020)^19,20^. All patients included in this study have given their informed consent. Enrollment occurred during the first epidemic wave in the period between March and August 2020. SARS-CoV-2 positive patients (n = 66) were selected on 3 clinical criteria: severe disease with a fatal outcome (n = 26), symptomatic disease with hospitalization and a favorable outcome (n = 22), asymptomatic disease with a positive RT-PCR test at inclusion (n = 18). The latter were most often contact cases of the former.

Patients from Madagascar were recruited as part of WHO’s First Few X cases (FFX) investigation protocol for coronavirus disease 2019^21^ approved by the Ethics Committee of Biomedical Research of the Ministry of Public Health of Madagascar (no. 058/MSANP/SG/AGMED/CERBM, March 30, 2020)^22,23^. Written informed consent was obtained from participants before enrolment in this study. For children and minors, written informed consent was obtained from parents or guardians on behalf of the minors enrolled in the study. All Malagasy patients included were SARS-CoV-2 RT-PCR positive and included asymptomatic individuals (n = 10) as well as patients exhibiting a symptomatic but non-lethal infection (n = 6). Malagasy pre-pandemic samples (n = 9) were obtained from a cross-sectional survey performed in 2015 and approved by the Ethics Committee of Biomedical Research of the Ministry of Public Health of Madagascar.

Overall, 123 individuals were included in this study, and allocated into 4 groups based on their infected status and their clinical outcome: uninfected pre-pandemic individuals (n = 41), asymptomatic infected individuals (n = 27), symptomatic infected patients (n = 23), and severe COVID-19 patients with fatal outcome (n = 32). None of the patient included in the study was affected by an active infectious disease such as HIV-1, Malaria or Tuberculosis. Epidemiological and clinical patients’ information were summarized in Table 1.

### Biological samples

Blood samples used for the serological analysis presented in this work were taken for all the patients within 2 weeks of their arrival at the hospital. The viral load was measured from nasopharyngeal swab. For the patients from the symptomatic and severe groups in Senegal, the median time lapse between the first reported symptoms and the blood sampling was respectively 11 and 13 days. In Senegal, additional blood samples were drawn from the patients in the course of disease to monitor parameters such as CRP and blood cell counts.

Plasma (EDTA blood) or serum samples from all recruited individuals were aliquoted and stored at − 20 °C or − 80 °C until used. The selected sera were shipped to JPT Peptide Technologies GmbH (Berlin, Germany) for peptide microarray analysis.

### Peptide microarrays

Peptide microarrays were specially designed by JPT peptide technologies GmbH (Berlin, Germany) as described previously^7^. Briefly, the peptides were synthesized using SPOT synthesis, cleaved from the solid support and chemoselectively immobilized on functionalized glass slides. Each peptide was immobilized on the microarray slides in triplicates as previously described^7^. The peptide library contained 487 overlapping 15-mer peptides with an overlap of 11 amino-acids. The peptides were derived from the 4 structural SARS-CoV-2 proteins E, M, N and S spanning the full antigen sequences: E (75 residues) covered by 16 peptides, M (222 residues) covered by 53 peptides, N (419 residues) covered by 102 peptides, S (1273 residues) covered by 316 peptides.

The peptide microarrays were incubated with sera (applied dilution 1:200) for 1 h at 30 °C, followed by incubation with 0.1 μg/mL fluorescently labelled anti-human-IgG (Jackson ImmunoResearch, 109–605-098) or anti-human-IgM (Thermo Fisher Scientific, A51012) detection antibody. Washing steps were performed prior to every incubation step with 0.5% Tween-20 in 1 × TBS. After the final incubation step the microarrays were washed again and dried in a microcentrifuge. Each microarray was scanned using a GenePix Autoloader 4300 SL50 (Molecular Devices, Pixel size: 10 μm). Signal intensities were evaluated using GenePix Pro 7.0 analysis software (Molecular Devices) and ranged between 0 and 60,000. All the epitope-mapping experiments were performed in the same Laboratory, using the same machines and the same buffers, by batches of 6 samples at a time.

### Data treatment

We used the software platform Amadea (ISoft, Saint Aubin, France) to treat the data. Amadea was initially developed in the Business Intelligence (BI) domain with the goal of extending collected data as well as reshaping, aggregating, and reformatting data in order to present it in a form that yields a better understanding and allows the end-user to make easier decisions. The Amadea Biopack (ISoft company) is one of the first tools from Business Intelligence which has been applied to Life Science^24^. Amadea software is based on Data Morphing technology, a high-performance engine that enhance discovery and decision making in research^25^. This platform is dedicated to the versatile exploitation of large-scale data, their rapid and straightforward analysis, allowing comparison between subgroups using any kind of test, with the immense advantage of being highly interactive to assess various parameters for an efficient and rapid guidance of the research.

The heatmaps obtained with the data from all the samples showed existence of “over-reacting” peptides and “over-reacting” sera. Negative controls (no serum) were used for batch effect control and to detect volatile reactions against some peptides. A few peptides (less than 5 per Ig type) exhibiting a high level of response in the negative controls (no serum) were deleted from the study. As for many large-scale studies, a standard correction was applied to modulate the non-specific part for sera overreacting against all peptides. To this effect, for each serum, 12.5 percentile of the anti-peptide responses was deleted from all peptide values (for a given Ig type). The rationale for these corrections was that a serum cannot be positive against all the peptides, therefore unnecessary background was decreased by deleting a certain percentile level per patient.

### Statistical comparisons

At first, a Student’s t-test with a Bonferroni correction was used to look for the peptides with higher responses in infected individuals than in uninfected ones (Table 2, results in bold). The Bonferroni correction was performed based on the number of independent tests that we evaluated at 487:3 = 162, since a linear epitope is approximately made of 4–6 amino-acids and may be thus covered by 3 consecutive peptides. The resulting threshold is thus 0.05:162 = 3 × 10^−4^.

The Student’s t-test corresponds to a comparison of means, and is thus not always adapted to detect positive values found at the top of a group compared to another one. We noticed that having the 25% top percentile twice as high in the infected patients as in the uninfected controls was a quite sensitive approach. To make this approach more robust and obtain a list of highly reliable hits, we used two additional criteria: signal levels of the 25% top percentile higher than 1000 in the infected group, and deleting the top signal to obtain the same results. This latter approach was used for the results presented in Tables 2 and 3.

We also defined a simple score of Ig response for each patient against each peptide. It corresponded to the Log_10_(corrected OD + 1), where corrected OD is the optical density of the sample obtained after data cleaning. This allowed us to compute a cumulative response score for each serum against the immunodominant peptides of Table 2. This Ig score was used to define 2 subgroups (higher than the median and lower than the median), and compare them for the main biological parameters (viral load, CRP, leucocyte counts) within the severe cases and within the symptomatic cases (Table 4). The same score was also used to measure the IgG response against the 13 potentially neutralizing peptides in the 4 groups of subjects (Fig. 3C).

The correlation between the IgG and IgM scores of individuals in each group was assessed using the Pearson correlation coefficient.

## Supplementary Information

Supplementary Information.

Supplementary Information.

## Data Availability

The data that support the findings of this study are available from the Institut Pasteur de Dakar or the Institut Pasteur de Madagascar but restrictions apply to the availability of these data, which are protected for ethical reasons, and so are not publicly available. De-identified data are however available through the following link http://griv.org/epimap/ (Accession number: EPiMAP-221). Additionally, the study protocol is available for request.
