# Supplementary material for: Linear epitope mapping of the humoral response against SARS-CoV-2 in two independent African cohorts
Source: Sci Rep. 2023 Jan 16;13:782. doi: 10.1038/s41598-023-27810-1 (PMC9842613; doi:10.1038/s41598-023-27810-1)
Supplement: Supplementary file 2 — Supplementary Information. [file 41598_2023_27810_MOESM2_ESM.pdf]

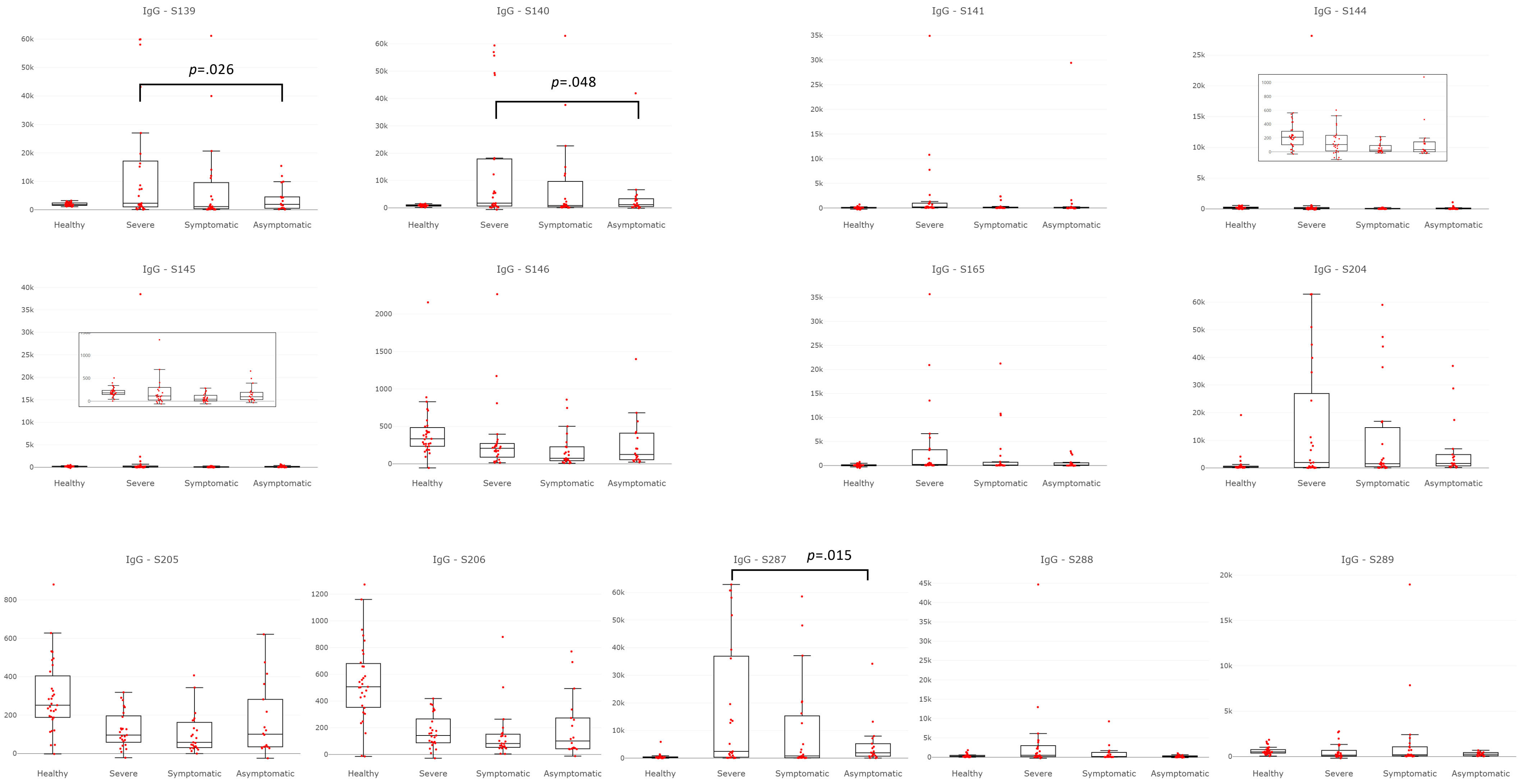
IgG - S139
p=.026
IgG - S140
p=.048
IgG - S141
IgG - S144
IgG - S145
IgG - S146
IgG - S165
IgG - S204
IgG - S205
IgG - S206
IgG - S287
p=.015
IgG - S288
IgG - S289
Healthy
Severe
Symptomatic
Asymptomatic

Supp Figure 1 Legend :

Box-plots presenting the IgG responses against the peptides covering the 4 previously published neutralizing regions (see text) in the uninfected, severe, symptomatic, and asymptomatic groups. P values are only shown for the comparison between 2 infected groups and given under the graph when significant. For peptides 144 and 145, a second graph with enlargement of the scale is provided.
